# Comparison of a healthy miRNome with melanoma patient miRNomes: are microRNAs suitable serum biomarkers for cancer?

**DOI:** 10.18632/oncotarget.3661

**Published:** 2015-03-26

**Authors:** Christiane Margue, Susanne Reinsbach, Demetra Philippidou, Nicolas Beaume, Casandra Walters, Jochen G. Schneider, Dorothée Nashan, Iris Behrmann, Stephanie Kreis

**Affiliations:** ^1^ Life Sciences Research Unit, University of Luxembourg, Luxembourg; ^2^ Klinikum Dortmund GmbH, Germany

**Keywords:** serum/tissue samples, circulating miRNAs, healthy miRNome, miRNA qPCR arrays, melanoma biomarkers

## Abstract

MiRNAs are increasingly recognized as biomarkers for the diagnosis of cancers where they are profiled from tumor tissue (intracellular miRNAs) or serum/plasma samples (extracellular miRNAs). To improve detection of reliable biomarkers from blood samples, we first compiled a healthy reference miRNome and established a well-controlled analysis pipeline allowing for standardized quantification of circulating miRNAs. Using whole miRNome and custom qPCR arrays, miRNA expression profiles were analyzed in 126 serum, whole blood and tissue samples of healthy volunteers and melanoma patients and in primary melanocyte and keratinocyte cell lines. We found characteristic signatures with excellent prognostic scores only in late stage but not in early stage melanoma patients. Upon comparison of melanoma tissue miRNomes with matching serum samples, several miRNAs were identified to be exclusively tissue-derived (miR-30b-5p, miR-374a-5p and others) while others had higher expression levels in serum (miR-3201 and miR-122-5p). Here we have compiled a healthy and widely applicable miRNome from serum samples and we provide strong evidence that levels of cell-free miRNAs only change significantly at later stages of melanoma progression, which has serious implications for miRNA biomarker studies in cancer.

## INTRODUCTION

A key prerequisite to any successful cancer therapy is its early diagnosis. In order to detect malignancies as early as possible, a large number of tailored laboratory tests for detection of marker proteins, metabolites, specific mutations and imaging of concerned body regions as well as biomarker profiling are routinely used in various clinical settings (Cancer Facts and, American Cancer Society, 2014).

Biomarkers are defined as “a characteristic that is objectively measured and evaluated as an indicator of normal biologic processes, pathogenic processes, or pharmacologic responses to a therapeutic intervention” [1]. As such, biomarkers are quantitative and robust measures that allow for unequivocal diagnosis of a specific disease, for assessing progression of disease or for monitoring response to treatment. The remarkable stability and tissue-specific expression profiles of miRNAs have raised hopes that these small non-coding nucleic acids could represent useful biomarkers that characteristically change their expression profiles in tissue and/or blood samples upon development and progression of cancer [2-4]. Since the first report about the potential of circulating miRNAs as biomarkers [5], many studies have reported cancer type-specific miRNA signatures [6-8].

Melanoma is one of few cancer entities with increasing case numbers almost anywhere in the world. Considerable therapeutical progress has been made in recent years with the introduction of specific kinase inhibitors targeting constitutively active BRAF present in more than 50% of melanoma patients. However, most such treated patients rapidly develop resistance against the drug requiring new and better therapies [9]. In a novel clinical approach, immunotherapies targeting immune checkpoint inhibitors CTLA-4 and PD-1 have been developed that show promising results in ongoing clinical trials [10]. However, the long term survival benefit for patients still remains to be established [11].

Given the clinical importance of melanoma, several research laboratories have ventured into quantification of miRNAs from blood, serum or plasma samples [12-17]. However, using different profiling platforms and inputs and variable techniques for quality control, normalisation and statistical evaluation, reported results show very limited congruence. Along these lines and regardless of the many publications claiming identification of specific miRNA biomarkers for cancer, several critical voices have recently summarised the technical and biological challenges of circulating miRNA profiling studies and they all together highlight the lack of consistency among published miRNA cancer signatures [6, 7, 18-25].

A major obstacle in miRNA profiling comes from technical issues related to the choice of platform and data analysis. Using qPCR array technology together with strict quality control measures at all steps of sample handling, data generation, processing and interpretation, we have compared serum-and tissue-derived miRNomes from healthy volunteers to melanoma patient samples representing all stages of disease.

Blood samples are an optimal source for detection of biomarkers. However, in order to achieve reproducible results, a healthy counterpart is necessary, representing a stable baseline against which changing miRNA profiles can be compared to. To our knowledge, no blood-based healthy miRNome is yet available in public databases comprising individuals from different ethnicities, gender and age. Here, we began to compile such a healthy miRNome, which can serve as a reference for secreted miRNA profiles to study melanoma or other cancers and diseases. A total of 52 melanoma patient serum samples and 30 healthy counterparts were analysed: statistically significant changes in the miRNome were only found at late stages of disease when advanced metastasis changes the integrity of body tissues. Furthermore, miRNA expression patterns were compared between melanoma tissues and blood samples, which revealed a subset of selectively secreted miRNAs.

## RESULTS AND DISCUSSION

### Study design and quality control

**Figure 1:**
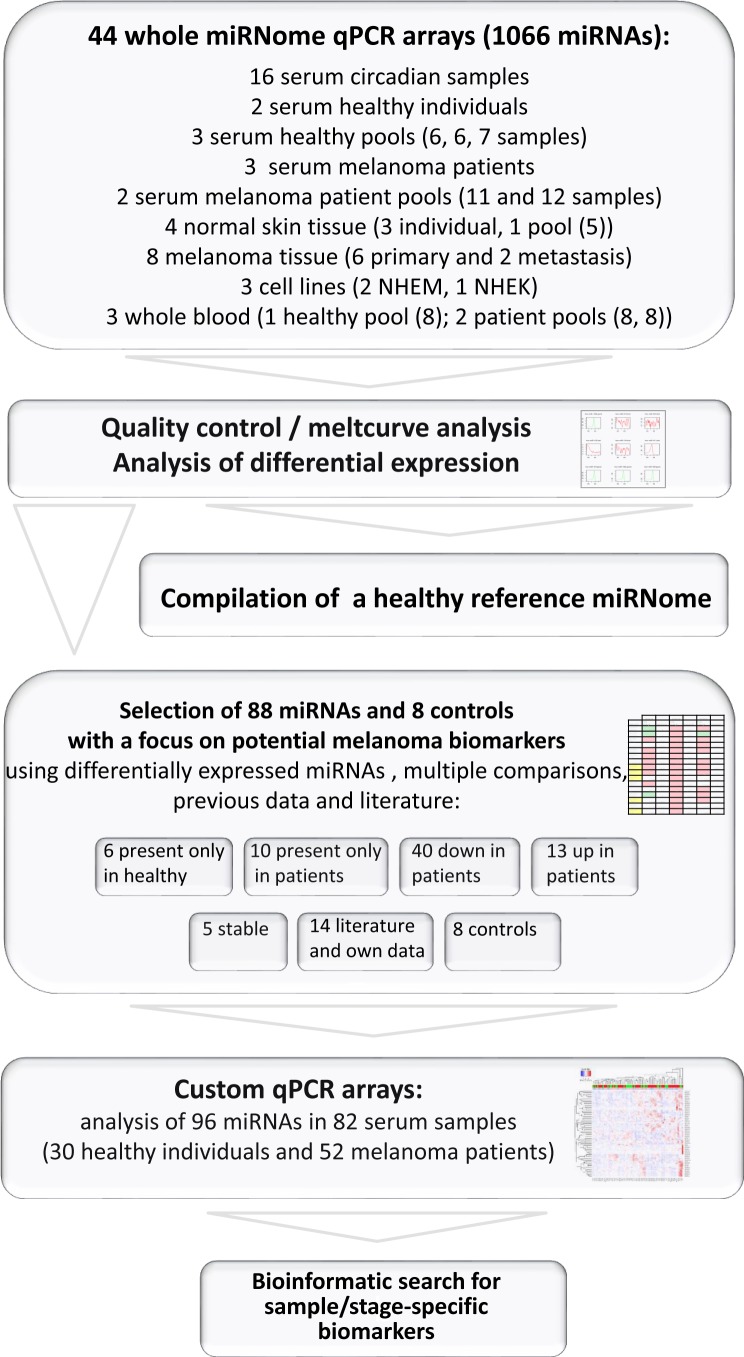
Overview of study design Numbers in brackets indicate number of separate samples included in pools. Individual melanoma patient serum samples analysed on custom qPCR arrays comprise 4 stage 0, 11 stage I, 17 stage II, 11 stage III and 9 stage IV samples.

Fig. 1 summarises the study design, the number and types of samples analysed by whole miRNome qPCR arrays and by customized qPCR arrays representing 88 miRNAs, selected to serve as potential biomarkers for the diagnosis of melanoma. Here, extensive quality control steps for sample collection, processing, data acquisition and analysis were applied in order to obtain robust and reproducible results, indicating whether secreted miRNAs could indeed be suitable biomarkers for melanoma. We chose a qPCR array platform with pre-amplification of mature miRNAs suitable for quantification of the typically low miRNA amounts in cell-free samples. Previous in-house data with hybridization microarrays and RNA-Seq revealed that qPCR would be the most useful technique for this purpose. In accordance, a recent comprehensive comparison of 12 commercially available miRNA profiling platforms also showed qPCR arrays to perform well in terms of reproducibly, sensitively and specifically quantifying low copy number miRNAs [26].

First, we compared sample types and found no major differences between serum and plasma (Supplementary Fig. S1A). To allow for inter-sample comparisons and to control for RNA extraction efficiency, we added exogenous RNAs (cel-39, cel-238 and cel-54) at different ratios (Supplementary Fig. S1B). Furthermore, RNA extraction protocols were optimized for precipitation of low miRNA amounts. RNA carriers introduced a bias and were therefore not used (Supplementary Fig. S1C). Cel-39 expression levels were used to calibrate data. Subsequently, we applied a protocol to control for potential contaminating miRNAs derived from platelets, lymphocytes and/or erythrocytes in each serum sample [27]. Samples showing hemolysis or the presence of contaminating blood cell-derived miRNAs were not considered for the study. Next, we established that pre-amplification was required to analyze all 1066 miRNAs by qPCR arrays (Supplementary Fig. S1D). Tests using selected miRNAs determined the general amplification factor between non- and pre-amplified templates to be 250-fold (Supplementary Fig. S1E). Additional experiments showed a good correlation between manual and whole miRNome qPCR (Supplementary Fig. S1F). Finally, a novel bioinformatic tool was developed allowing for rapid analysis of thousands of melting curves, a necessary quality control step for SYBR-green based qPCR amplifications (which is often omitted in many published studies) before analysis of differential expression can be performed. MiRNAs with bad melt curves, indicating unspecific amplification despite sometimes low Cq values, were disregarded.

Due to the lack of stable reference miRNAs in serum samples, data normalisation and analysis pose a challenge. Noteworthy, miR-16-5p and RNU6B, which are regularly used for normalisation of miRNA expression levels in cell-free samples [6, 28] were either unstable (especially in slightly hemolytic samples, miR-16-5p) or absent (RNU6-2) in our data sets (data not shown). In Materials and Methods, a detailed description of the different approaches for data analysis is provided. Taken together, we invested considerable effort to set up an analysis pipeline that allows for sensitive and specific quantification of miRNAs in cell-free samples.

### The healthy miRNome

**Figure 2:**
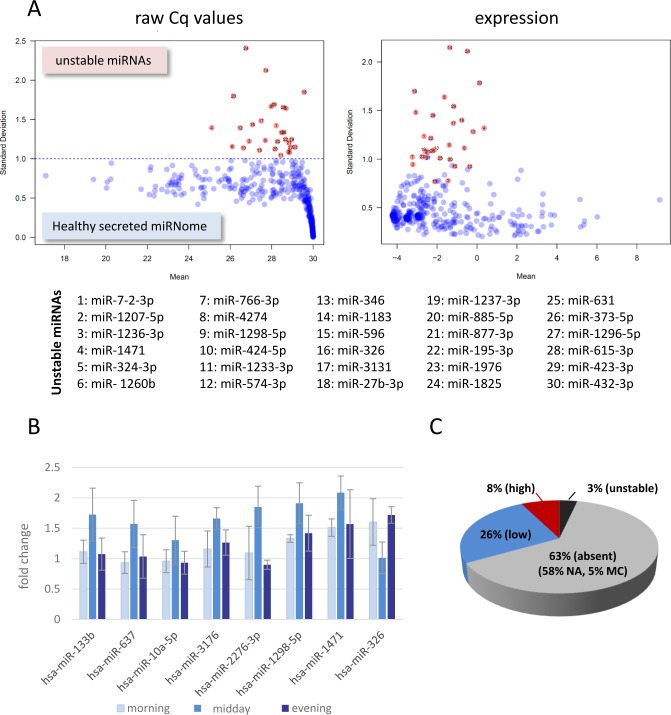
The secreted healthy miRNome A) Stability of the healthy miRNome. Mean-standard deviation plots of averaged raw Cq values or normalized expression for 1066 miRNAs (qPCR arrays of 3 serum pools (from 19 individual healthy donors) and circadian serum samples from 4 individuals). 30 miRNAs (list) with a STD above 1 for Cq values (red dots) were determined as unstable. MiRNAs absent in all samples as well as the averaged healthy miRNome are shown in Supplementary Table S2. B) Analysis of circadian miRNomes. Twelve serum samples of 4 volunteers collected at different times of day were compared to the healthy miRNome. Fold changes +/− SD were calculated for miRNAs that had a measurable higher or lower expression at one time point only (midday). C) Pie chart summarising data of the healthy miRNomes. NA: not expressed (Cq values >30 in all samples), MC: bad melt curves in more than 80% of samples, low expression: Cq values >27 in more than 80% of samples, high expression: Cq < 27 in more than 80% of samples, unstable: list of Fig. 2A.

Given the lack of suitable small reference RNAs in cell-free samples and considering that most miRNA expression changes are notoriously small, a standardized healthy miRNome serving as a “baseline control” for all profiling studies is essential. Similar to the reference genome, which is composed of sequences from different individuals, we have started to assemble a healthy miRNome, using serum samples from 23 healthy individuals including 4 volunteers where blood samples were drawn at different times of day (circadian sample set). MiRNome data were collected by qPCR arrays and expression values for each miRNA were calculated with the aim to detect miRNAs that fluctuate between healthy individuals, of which we found 30 (3% of 1066 analysed miRNAs, Fig. 2A). These fluctuating miRNAs should be interpreted with care if identified in any kind of differential miRNA expression analysis.

In order to control for possible effects that the time of sample collection could have on the stability of the miRNome, samples were collected at different times of day (circadian samples). No major expression changes were identified when we looked for miRNAs that showed higher expression at one time point only. Only 7 miRNAs (0.9% of 1066, Fig. 2B) showed modest augmentation after lunch suggesting that these could be potential circadian and/or metabolic miRNA candidates associated with uptake of food. The lack of circadian cycle expression changes in circulating miRNAs was intriguing and similar to what has recently been described by Keller and colleagues [29]. On the other hand, miRNAs have been shown to post-transcriptionally regulate expression levels of circadian clock genes and change with age [30-32].

In summary, we compiled a reference data set (Supplementary Table S2) representing a remarkably stable secreted cell-free miRNome. Sixty-three % of miRNAs were not expressed in serum samples, 3% fluctuated between individuals and only 8% (82 miRNAs with a Cq value below 27 in 80% of samples) showed high expression values in serum samples (Fig. 2C). To make the healthy secreted miRNome as robust as possible we are continuing to collect samples for the healthy cohort.

### miRNA profiling of melanoma samples

**Figure 3:**
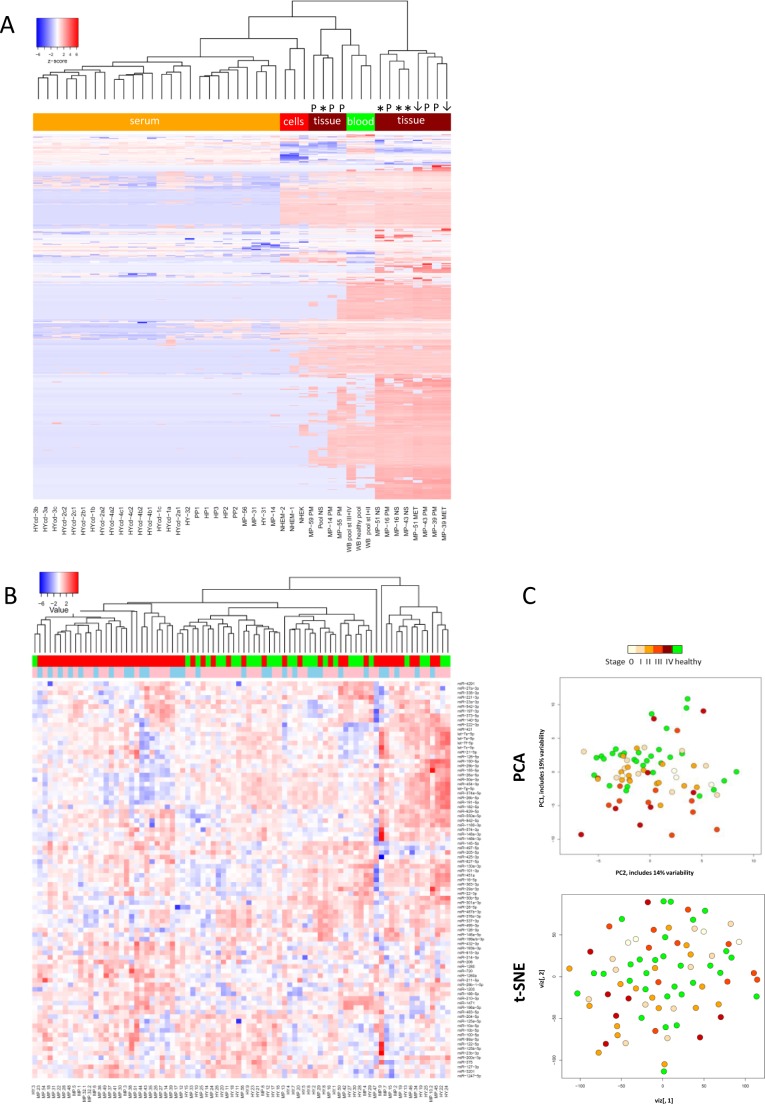
MiRNA profiling of melanoma samples A) Heatmap of whole miRNome qPCR array data of all 44 samples (see Fig. 1) normalized by global plate mean (miRNAs absent across all samples are not depicted). WB: whole blood, NS: normal skin tissue, MET: metastatic tissue, PM: primary melanoma tissue, st: stage; NHEK: normal human epidermal keratinocyte cell line; NHEM: normal human epidermal melanocyte cell line, HY: healthy, HYcd: healthy circadian, MP: melanoma patient, HP: healthy pool, PP: patient pool. * represent normal skin, arrows metastatic tissue and “P” indicates primary tumour. B) Heatmap and C) PCA and t-SNE plots of 82 individual serum samples (30 healthy volunteers and 52 melanoma patients) analysed by custom qPCR arrays. Data were normalized using the 5 most stable miRNAs. Colours: blue-male; pink-female; green-healthy; red-patient.

The heatmap in Fig. 3A shows an overview of whole miRNome data sets for melanoma tissue samples (discussed below), normal human primary melanocytes (NHEM) and keratinocytes (NHEK) as well as for healthy individuals and melanoma patient serum samples. Three whole blood sample pools representing healthy, early and late stage melanoma patients have remarkably similar expression patterns while they were clearly distinct from serum samples. Overall, normal skin, primary lesion and metastatic melanoma tissue samples were well discernible. Of note, two of the primary melanoma tissues (MP-39.PM and MP-43.PM) resemble the only two metastatic melanoma samples included here (MP-51.MET and MP-39.MET) possibly suggesting a more advanced tumor stage. Skin tissue samples always contain a varying amount of keratinocytes. To account for the intrinsic heterogeneity of tissue samples, we also analysed primary cell lines of keratinocytes (NHEK) and melanocytes (NHEM), which show discrete expression levels for several miRNAs and this could help to allocate expression profiles of individual miRNAs from melanoma tissue samples to the respective cell types. Overall, the whole miRNome data separate sample types as such that whole blood samples, primary cell lines, tissue and serum samples show distinct expression patterns with the expression levels of most miRNAs being very low or absent in serum samples (also see Fig. 2C). Therefore, we opted for custom-made qPCR arrays that contain only the most interesting miRNAs for the current study. Fig. 3B depicts relative expressions of 87 miRNAs (excluding miR-374c-5p that was absent in all samples) in 82 serum samples (30 healthy-green; 52 melanoma-red; 52 female-pink; 30 male-blue). The heatmap, PCA and t-SNE plots (Fig. 3C) illustrate that no clustering according to healthy versus melanoma or gender was evident. Overall, variations in expression levels of individual miRNAs were relatively low with few exceptions (e.g. miR-127-3p, miR-337-3p).

To confirm the absence of clustering according to gender, we averaged the expression values of all miRNAs on the custom arrays for female and male samples (Supplementary Fig. S2A). Next, we separated the data sets into healthy and melanoma samples. Supplementary Fig. S2B reveals that the healthy miRNomes display similar expression profiles for most miRNAs. In contrast, the 52 melanoma serum samples show a more or less random distribution with no obvious grouping according to disease stage (Supplementary Fig. S2C).

**Figure 4:**
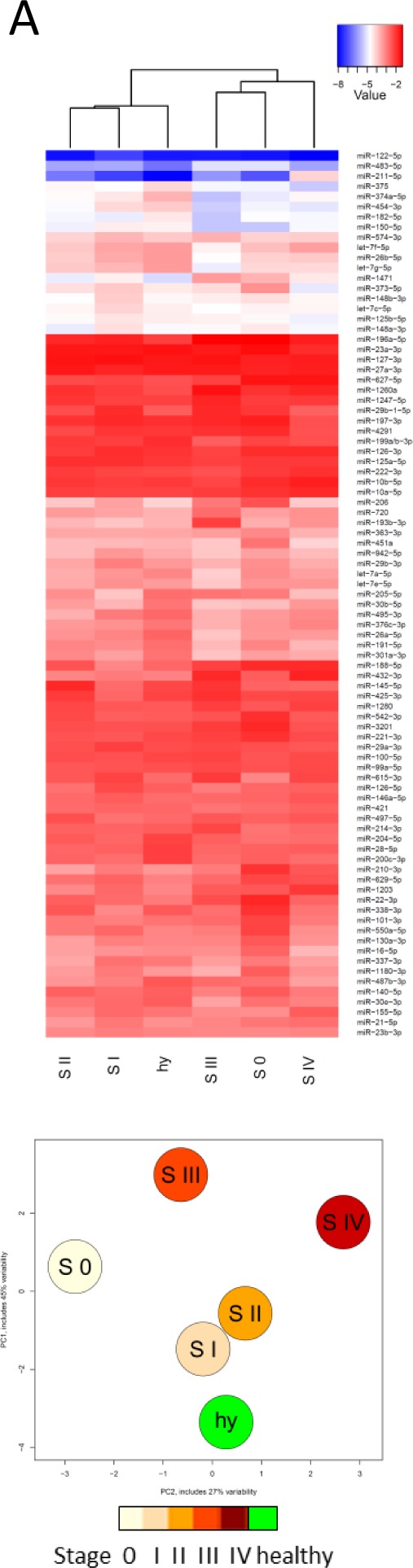

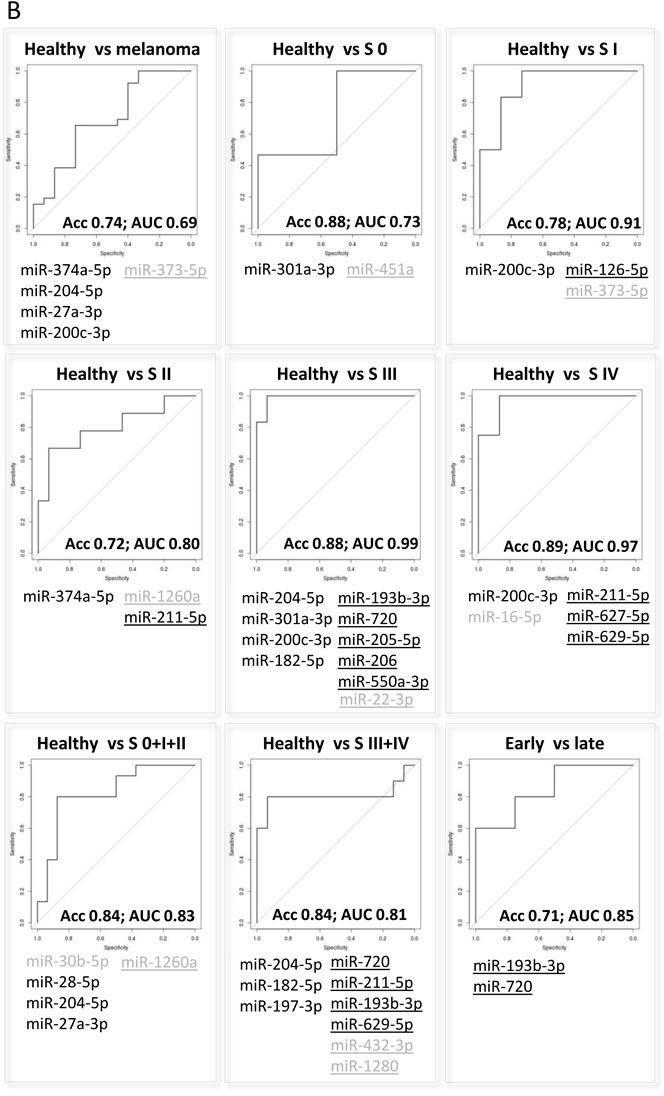
Biomarker studies A) Heatmap and PCA showing the averages of samples belonging to different melanoma stages and healthy serum samples (based on Fig. 3B and 3C). S: stage; hy: healthy. B) ROC curves on pairwise comparisons between indicated sample groups (30 healthy, 4 stage 0, 11 stage I, 17 stage II, 11 stage III, 9 stage IV samples; early: stage 0+I+II; late: stage III+IV). Underlined miRNAs were upregulated while all others were downregulated compared to healthy or early stage samples. MiRNAs in grey shade should not be considered as potential biomarkers because they were highly expressed (Cq < 15) in whole blood samples and might therefore derive from contaminating blood cells (miR-1260a, miR-22-3p, miR-1280, miR-451a and miR-16-5p, also see Supplementary Table S3), or they were part of the unstable miRNAs (miR-432-3p, miR-373-5p; see Fig. 2A). ACC: accuracy value, AUC: area under the curve.

To nevertheless allow for identification of potential disease stage-specific miRNAs, we combined these samples according to their cancer stage and compared them to the average of the healthy samples (Fig. 4A). The different groups revealed distinct expression patterns, which prompted us to find signature miRNAs, potentially characteristic for the different stages of melanoma. First, accuracy values were computed to identify the optimal number of miRNAs required to make correct stage assignments. Then, AUC values were calculated for the different groups (Fig. 4B). Results from heatmaps and ROC curve analysis correlated well. Fig. 4B shows that upon comparison of healthy individuals with stage III or IV metastatic melanoma samples, biomarkers were found with excellent predictive scores (AUC 0.99/CI 0.96-1.00 for stage III and AUC 0.97/CI 0.89-1.00 for stage IV). Several miRNAs were profoundly down-regulated in late stage melanoma patients (e.g. miR-200c-3p, -204-5p, -182-5p, -301a-3p) while others were up-regulated (e.g. miR-211-5p, -193b-3p, -720, -205-5p). Six of these potential biomarkers were also described to be differentially regulated in secreted melanoma exosomes compared to healthy melanocytes [33]. Those identified miRNAs contain several new candidates as well as some miRNAs that have previously been connected to melanoma. MiR-211-5p is overexpressed in the plasma of late stage melanoma patients [15]. In this context, we have recently shown that miR-211-5p has no evident role in inhibiting invasion of melanoma cells [34] as was claimed before [35-37] and we describe here that indeed miR-211-5p is highly overexpressed in serum of stage IV melanoma patients when compared to healthy controls. In fact, the overexpression levels (68-fold) of this miRNA in serum samples of stage IV melanoma patients were the highest measured throughout the study (data not shown). MiR-211-5p has long been recognised as a marker miRNA for the melanocytic lineage [38, 39] and its high levels in sera of late stage patients might suggest that it is specifically secreted from melanoma cells.

Given the clinical importance of melanoma, several miRNA quantification studies have been performed using tissue, blood, serum or plasma samples in order to identify biomarkers that could diagnose or predict disease. Greenberg et al. reported miR-29c-5p and miR-324-3p to be lower in the serum of metastatic melanoma patients (stage IV) compared to healthy control individuals [16]. Both miRNAs could discriminate between melanoma and colon or renal cancer. A signature of 5 serum miRNAs (miR-150-5p, -15b-5p, -199a-5p, -33a-5p, -424-5p) was described as biomarkers for recurrence in melanoma [13] while Leidinger and colleagues suggested a signature of 16 miRNAs from whole blood samples that could be used to separate melanoma from healthy control samples [14]. We found no overlap with our data on whole blood samples probably because different platforms and methodologies were used (Supplementary Table S3). In another report, the oncomiR-21 was found highly expressed in melanoma patient plasma matching high expression levels of this miRNA in melanoma tissues [15]. Own previous data indicated reduced expression levels of miR-200c-3p and miR-205-5p in metastatic melanoma tissue samples [40]. Levels of miR-182-5p, important for melanoma progression, had not been analysed before in serum and are shown here to be lower in stage III patients compared to healthy volunteers.

The usefulness of biomarkers should be determined not only by their sensitivity and specificity but also by their clinical relevance [41]. In case of melanoma, the clinical importance of markers for the diagnosis of the malignancy is limited as the vast majority of cases can readily be diagnosed by visual inspection through a dermatologist followed by histological confirmation of excised tissue material [42]. However, at very early stages of disease or when primary lesions are occult (or during BRAF kinase inhibitor treatments), a biomarker test indicative of early stage cancer or development of drug resistance would be clinically valuable. Here, serum samples from patients with metastatic melanoma showed marked expression changes in some miRNAs that could qualify as biomarkers only at late stages of disease. However, when rigid quality control measures were applied, a reliable distinction between the healthy miRNome and early stage melanoma or melanoma patients overall was not possible.

In agreement with the recently voiced critical thoughts [18, 21], the scientific community will have to agree on quality standards and possibly personal cutoffs and thresholds [43] for profiling miRNomes and quantifying individual miRNAs (similar to the MIAME and MIQE rules for microarray and qPCR data, respectively) if we are to identify sustainable miRNA biomarkers. Otherwise, data from different laboratories and studies will remain almost impossible to compare and only by adding more data sets, we will not accomplish the goal of finding suitable and reliable biomarkers for cancer and other diseases.

### Comparison of circulating miRNAs with matching tissue

**Figure 5:**
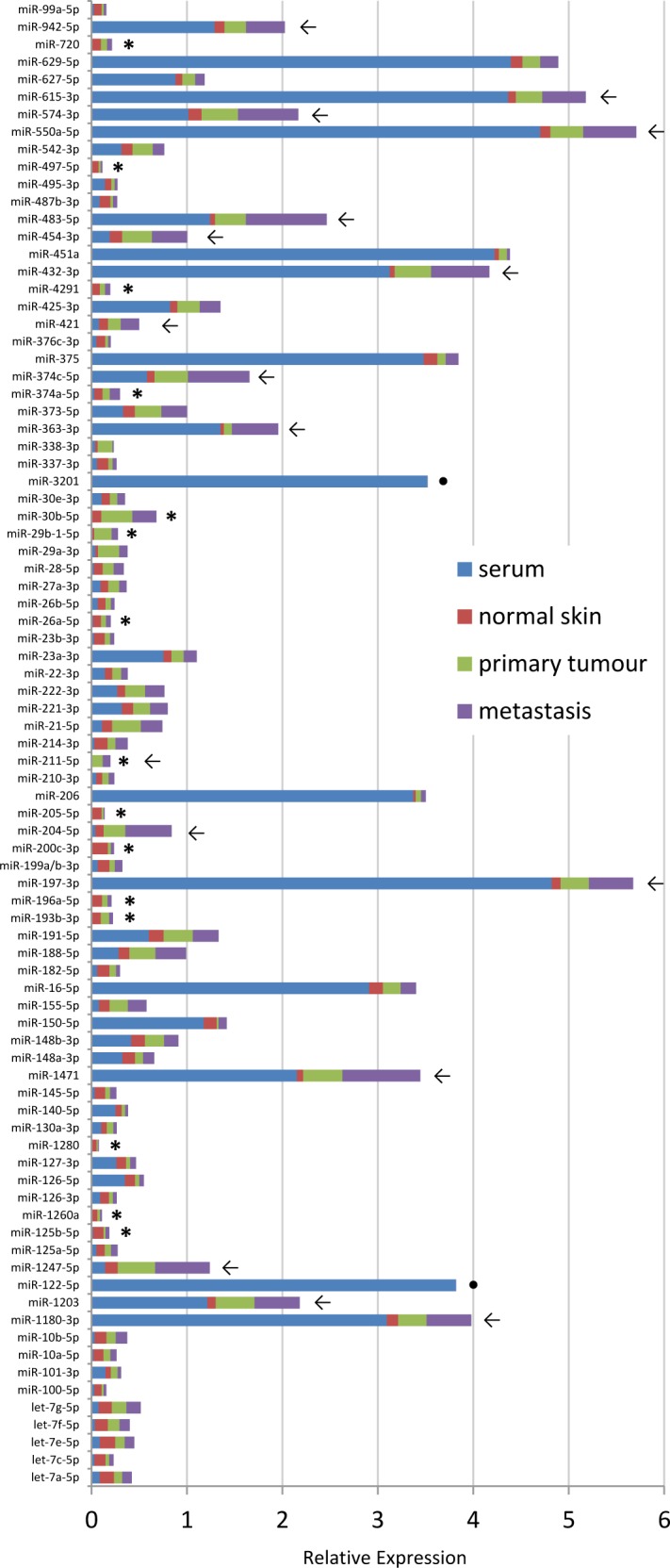
Comparison of tissue and serum samples Bar diagram showing averaged relative expression values from available serum (blue), normal skin (red), primary tumour lesion (green) and metastatic lesions (purple) samples. Arrows indicate gradually increasing expression of miRNA with progression of disease, stars highlight miRNAs that were only detected in tissue samples but not in serum, dots mark miRNAs with serum expression only. Expression levels of single samples can be seen in Supplementary Fig. S3.

To investigate differences in miRNA expression related to the type of sample, we compared serum-and tissue-derived miRNomes from 4 individuals and found several miRNAs (of the 88 candidates on custom plates) with distinct intracellular and extracellular levels (Supplementary Fig. S3). Fig. 5 depicts averaged expression levels for available serum, normal skin, primary tumour and metastasis samples. Noteworthy, direct and accurate comparisons between miRNA expression levels in serum and tissue samples remain problematic because of the very low RNA amounts in serum samples that are difficult to quantify while known and standardized amounts are used for amplification from tissues. On the other hand, evaluation of same sample types amongst individuals is feasible and the whole miRNome heatmap (Fig. 3A) shows that the overall expression levels of many miRNAs was increased in tissue samples from late stage melanoma patients compared to healthy individuals, normal skin samples and primary tumours.

Fifteen miRNAs (17%) were exclusively found intracellularly (marked with a star in Fig. 5) and several miRNAs (such as miR-204-5p, -211-5p, 374c-5p, -363-3p, -483-5p) show a gradual increase in tissue expression levels with progression of disease (normal skin → primary lesion → metastasis; marked with an arrow). In line with previous studies, miR-200c-3p and -205-5p were only observed in normal skin but hardly in tumour samples [40, 44]. Similarly, members of the miR-29 family had higher levels in primary tumours than in metastatic samples indicating potential tumour-suppressive roles for these miRNAs as was described before [45, 46]. MiR-211-5p and miR-204-5p belong to the same family sharing most of their target genes and have previously been assigned key roles in melanoma development [36, 47]. Here, we also detected both miRNAs in melanoma tumour tissues. It was recently stated that expression levels of a given miRNA should correlate between tissue and matching blood samples so that high levels of a miRNA in circulation should be matched by high levels of this miRNA in the respective tissue [18]. However, another scenario is also likely in which cancer cells specifically, randomly or by unknown processes completely secrete certain miRNAs at a given time point. In this case, miRNA levels in tissue and in cell-free preparations could be inversely correlated. Moreover, serum miRNAs may derive from other tissues or organs not necessarily related to the tumour. We found 12 cases (of 88) where miRNAs had >3-fold higher expression level in serum than in tissue and 2 miRNAs were exclusively detected in serum (miR-3201 (unknown function), miR-122-5p (liver-specific), marked with a dot in Fig. 5).

In conclusion, expression profiles for the majority of analysed miRNAs in tissue and serum samples from the same individuals were similar to each other with only 16% of miRNAs reaching considerably higher relative levels intracellulary than in circulation. Several interesting miRNAs have been identified that increase their expression levels with stage of disease. However, most of those were not detected as serum-based biomarker candidates for late stage melanoma patients described above, which once again highlights the difficulty to identify robust blood-based miRNA biomarkers (also see Fig. 4).

The underlying idea of measuring secreted miRNAs in cell-free samples is that the presence of cancer (or other diseases) induces changes in the levels of secretion so that different amounts and profiles of miRNAs in circulation can be translated back to a disease or response to treatment. Whether cellular transformation requires the active shuttling of miRNAs or whether it is a coincidental “by-product” of malignancy (released by apoptotic cells) and which miRNAs are preferentially secreted via exosomes or bound to proteins such as Ago2, is currently unknown. Several studies have demonstrated that secreted miRNAs can act as intercellular messengers [48-50] and although the molecular events are incompletely understood, it is tempting to speculate that upon cancer formation, secreted miRNAs may play a role in establishing continued growth or even metastasis distant from the primary lesion. If this is the case, specific intracellular and extracellular patterns of miRNAs should be detectable that correspond to different cancer entities and stages provided that no other confounding diseases blur the picture. MiRNAs, that are disease-specifically secreted (via exosomes or bound to Ago2) into the blood may convey signals to recipient cells, where the engulfed circulating miRNA could then, together with endogenously expressed miRNAs, down-regulate target mRNAs and in doing so participate in pathological events. However, only few studies have so far demonstrated such a biological function of circulating miRNAs [49-52].

Apart from expression profiles of secreted miRNAs, we also began to investigate the potential functional role of such circulating miRNAs. In a first set of experiments we isolated exosomes from patients' sera and proved that certain miRNAs (here miR-211) were highly expressed. Those miRNA-containing exosomes were then transferred to A375 melanoma recipient cells, which are devoid of miR-211. Surprisingly, several previously confirmed target genes of miR-211 [34] were not found to be down-regulated in those cells indicating that the amount of exosome-transferrable circulating miRNAs might not be sufficient to significantly reduce mRNA expression levels of target genes in recipient cells (data not shown). While this finding requires further confirmation with more miRNAs and target genes, it nevertheless provides a first indication that the assumed functional role of secreted miRNAs as specific paracrine messengers might have to be revisited.

Altogether, our and previous results show that several miRNAs are actively shuttled out of cells under certain conditions (possibly because the cell has so far unidentified mechanisms to rid itself of miRNAs). It remains to be shown though whether secreted miRNAs could actively partake in transferring signals or in functionally down-regulating target mRNAs by overwriting or supporting the endogenous miRNAs in other target cells. Many factors can confound the levels of circulating miRNAs and indeed it remains to be seen whether we will soon have developed commonly applied sampling, processing and profiling protocols that can clearly discriminate between truly secreted miRNAs indicative of malignant processes from contaminating miRNAs derived from platelets, erythrocytes, lymphocytes or normal cell death. In a next step, it needs to be established whether such truly secreted miRNAs, once we have means to reliably quantify them, may have biological functions in intercellular communication.

## MATERIALS AND METHODS

### Patient samples

Serum and whole blood samples, primary and metastatic melanoma tissue and corresponding healthy skin samples were collected at the Dermatology Unit of the University Hospital in Freiburg, the Dermatology Clinic in Dortmund, at the University Hospital in Homburg (all in Germany) and at the Red Cross in Luxembourg. Circadian samples from healthy individuals were collected in the morning (before breakfast), at midday (after lunch) and in the early evening (before dinner). Sample collection was approved by the respective ethics committees in Germany and Luxembourg and written informed consent was obtained from all healthy controls and patients. Further information on samples is given in Supplementary Table S1.

### Study design

Samples were profiled using whole miRNome qPCR arrays (Qiagen, miRBase v.16 with 1066 miRNAs). In a first step, groups of healthy donors and melanoma patient serum samples (representing early or late stage melanomas) were pooled to average out individual variances and to find robust differences between populations or were analysed individually (Fig. 1). From this analysis, 88 miRNAs were selected to be spotted on customized qPCR arrays (Qiagen, Germany) taking into consideration miRNAs differentially expressed between healthy and disease samples, several stable miRNAs, previous own data as well as interesting miRNAs described in literature (Fig. 1 and Supplementary Table S2). A further 82 serum samples were subsequently analysed on such custom qPCR miRNA arrays. Additionally, qPCR data derived from healthy serum samples were used to establish a secreted healthy reference miRNome (Fig. 2 and Supplementary Table S2).

### Quality control

The lack of consistent protocols for circulating miRNA profiling and the absence of stable secreted reference miRNAs leads to a large variability and incoherent results reported in different studies. Recent publications have highlighted some of the technical problems of miRNA biomarker studies [18, 21, 23]. We have addressed most of these issues and combined several measures to ensure high quality and reproducibility of our data:

• Standardised protocols for sample collection and processing based on previous testing of different centrifugation steps, tubes, sample types (serum or plasma) (data not shown and Supplementary Fig. S1A).

• Standardised protocols for RNA extraction based on previous optimisation of different sample input volumes (data not shown) and use of RNA carriers (Roche). As shown in Supplementary Fig. S1C, RNA carriers introduced a bias and were therefore not used in this study.

• Spike-in controls of 3 exogenous *C.elegans* miRNAs (cel-39, cel-54, cel-238) [25] in 3 different concentrations were added to account for biases in quantification of miRNAs with low or high abundance and to control for quality of the serum sample RNA extraction (different spike-in ratios were previously tested, Supplementary Fig. S1B). Cel-39 expression was also analysed on the whole miRNome and custom qPCR arrays and its expression values were used to calibrate data for all serum samples. We are aware that spiked-in RNAs are not the perfect controls for the efficiency of RNA extraction [53, 54] but together with other controls (see below) it currently represents the best possible way to control miRNA quantification results.

• Thorough quality control RT-qPCRs were performed on each serum sample prior to analysis on qPCR arrays using all of the following primers: cel-39, cel-54, cel-238, miR-451a, miR-23a-5p, SNORD61, SNORD68, SNORD72, SNORD95, SNORD96A and RNU6-2 (details under “RNA extraction and RNA quality control). Samples not meeting QC standards were excluded from the study.

• Qiagen qPCR arrays with optional pre-amplification were chosen as they have high quality scores compared to 11 other platforms [26]. The necessity of pre-amplification was established by comparing the positive calls on the qPCR arrays with and without this step (Supplementary Fig. S1D). Further, the introduced amplification factor was determined for specific miRNAs (Supplementary Fig. S1E). The qPCR arrays have default miRTC (internal reverse transcription control) and PPC (positive PCR control) spotted on each plate. Only plates with correct values for all internal controls were used for follow-up analysis.

• We compared the amplification efficiency of Qiagen miRNA qPCR arrays with manual qPCR amplifications for 11 selected primers (Supplementary Fig. S1F) and found a high correlation of results.

• Due to the absence of well-expressed and suitable reference miRNAs in serum that could be used for normalisation, we applied different normalisation methods. They were based either on means of commonly expressed miRNAs (“global mean method”, for whole miRNome qPCR arrays) or on “RefFinder” (for custom miRNA arrays), a webtool, which integrates results from geNorm, Normfinder, and BestKeeper as well as the comparative ΔΔCt method to determine the 5 most stable miRNAs in each data set (http://www.leonxie.com/referencegene.php).

• A healthy serum miRNome was compiled to allow for better comparison with cancer samples (Fig. 2 and Supplementary Table S2).

• Since there is not much known about the consistency or differences of miRNAs expressed in tissue versus circulation, we compared in 4 individuals patterns of circulating miRNAs to their tissue samples (Fig. 5 and Supplementary Fig. S3). We tried to quantify RNA extracted from serum using Nanodrop (ThermoScientific) and HighSens quantification chips (BioRad) but had no consistent results. Therefore, the amount of input material for reverse transcription from serum samples may vary and be less consistent than input amounts from tissue-derived samples where RNA quantification is possible.

• A total of 126 samples (melanoma and healthy controls, whole miRNome and custom profiling) were used as well as more miRNAs than are usually tested (starting from whole miRNomes, with 1066 miRNAs v.16 down to 88 selected miRNAs on custom plates).

### Sample collection

In order to minimize variability derived from sample collection and handling, a standard procedure was developed that was strictly adhered to for all sample collection and processing steps. After blood withdrawal, serum tubes were left at room temperature for 1 hour; samples were then centrifuged for 15 min at 2000 rpm (750 g) at room temperature. Subsequently the serum was removed, aliquoted, snap-frozen and stored at −80°C until RNA extraction. Whole blood was collected in PAXgene Blood RNA tubes (BD Biosciences) according to the manufacturer's instructions and stored at −80°C until RNA extraction. Primary melanoma, melanoma metastasis, as well as the corresponding healthy skin were excised and immediately stored at –80°C until further use. Staging of diseases was performed by the histology departments of the respective clinics.

### Total RNA extraction and RNA quality control

To extract total RNA from whole blood, the PAXgene blood miRNA kit (PreAnalytiX) was used. Total RNA from NHEM, NHEK and A375 cells was extracted using the miRNeasy Mini kit according to the manufacturer's instructions. Tissues (primary and metastatic melanoma and healthy skin) were lysed in RLT Plus buffer with the TissueLyser LT (Qiagen) and total RNA was extracted with AllPrep DNA/RNA Mini kit (Qiagen) according to the manufacturer's instructions. Eluted RNA was subsequently processed by standard ethanol precipitation. Quantity and purity of whole blood, tissue and cell line RNA were assessed using a Nanodrop ND-2000 Spectrophotometer.

Total RNA from 200 μl serum was extracted using the miRNeasy serum/plasma kit (Qiagen) according to the manufacturer's instructions. Before the addition of Qiazol, the serum was thawn at room temperature and centrifuged for 30 min at 13000 rpm (16000 g) at 4°C to remove cellular debris. As an internal calibrator, a mix of cel-39, cel-54 and cel-238 exogenous controls was spiked into the samples. RNA was eluted with 14 μl of RNase-free water. Quality control of serum RNA was performed by RT-qPCR using primers for:

• Cel-39, cel-54, cel-238 spikes to control for variations in recovery and amplification efficiency between RNA preparations

• miR-451a (highly expressed in blood cells, [19, 55]) and miR-23a-5p (relatively stable in serum and not affected by hemolysis) to calculate a hemolysis indicator [27]

• SNORD61, SNORD68, SNORD72, SNORD95, SNORD96A and RNU6-2 highly expressed in blood cells and normally absent in serum to control for blood cell contamination of the serum (Qiagen White paper, Shaffer et al., 2012).

Briefly, 4 μl out of 12 μl eluted total RNA from 200 μl serum were reverse transcribed in a 10 μl reaction volume with the miScript RT II kit (Qiagen) following the supplied protocol using Hispec buffer, which specifically amplifies only mature miRNAs. Real-time PCR detection of the above-mentioned mature miRNAs was carried out on a CFX96 Detection System (Bio-Rad) using 1 μl of 1:10 diluted cDNA, 2x iQ SYBR Green Supermix (Bio-Rad) and 10× miRNA-specific primer assay (Qiagen). Specificity of the qPCR primers was assessed by a post-qPCR melting curve analysis. All serum samples were quality controlled in the above described way. Samples not reaching sufficient quality metrics due to hemolysis, white blood cell contamination or incomplete recovery of spiked-in controls were excluded from the study (31 of a total of 131 serum samples).

### miRNA profiling by qPCR arrays

Due to generally low amounts of miRNAs in serum samples, we opted for a pre-amplification step for all samples (see Supplementary Fig. S1D). MiRNAs of serum, whole blood, tissues and cell lines were profiled with human whole miRNome miScript miRNA qPCR arrays (Qiagen, v.16, 1066 miRNAs) or on custom qPCR arrays (Qiagen). For serum, 4 μl out of 12 μl eluted total RNA from 200 μl serum, and for tissue, whole blood and cell lines 50 or 100 ng RNA were reverse transcribed in a 10 μl reaction volume with the miScript RT II kit (Qiagen) using Hispec buffer. Regarding serum pools, corresponding RNA samples were pooled and 4 μl of pooled total RNA were reverse transcribed as described above. The 1:5 diluted cDNA was pre-amplified with the miScript PreAMP PCR kit (Qiagen) using the corresponding primer mixes (whole miRNome primer mix for whole miRNome qPCR arrays and custom primer mix for custom qPCR arrays). Pre-amplification control experiments were performed by RT-qPCR using primer assays for miR-16-5p, SNORD95, cel-39 and miRTC (internal miRNA reverse transcription control). Quality controlled pre-amplified cDNA was diluted 1:5 and further used for miScript whole miRNome and custom qPCR arrays (Qiagen). All kits and qPCR arrays were used according to the supplied protocols. Real-time PCR detection on the qPCR arrays was carried out on a CFX384 Detection System (Bio-Rad). Specificity of the qPCR primers was assessed by a post-qPCR melting curve analysis (see below). The selection process of primer assays to be spotted on the custom qPCR arrays is described in the Methods section and in Fig. 1. Raw data files of all qPCR arrays are available upon request.

### Data analysis

For qPCR array data analysis, baselines and thresholds were adjusted as recommended by the supplier and Cq values were exported for analysis. Cq values obtained with the cel-39 primers were used to calibrate the data sets: the Cq mean for cel-39 for each sample was calculated, the highest Cq mean of all samples was determined and the difference (correction factor) with the other samples was established. This correction factor was added to all Cq values of a sample. Calibrated Cq values greater than 30, as well as primers with bad melting curves (see below) were considered as not detected (N/A). These lower cut-off Cq values, recommended by the supplier, are due to the additional 12 PCR cycles during pre-amplification. Because of the lack of established house-keeping genes in serum imperative for data normalization, we used means of commonly expressed miRNAs (for whole miRNome qPCR arrays) or the 5 most stable miRNAs individually determined by RefFinder (see above).

### Quality control of qPCR melting curves

To assess the quality of melting curves from the whole miRNome qPCRs, we have developed a Support Vector Machine (SVM)-based method based on the R ‘e1071’ package, which is available upon request. Our tool is able to recognize “good” and “bad” curves, based on their shapes and curve features such as the Cq values, the melting temperatures, the peak heights, and the starting and end temperatures. All information was extracted from the Biorad run files from which post run melting curves were obtained. 6847 manually annotated and controlled curves were used as a training set. To assess the efficiency of this method, one third (2281) of this set was removed before training. Then the ROC score was computed for this training/test set combination. This process was repeated 100 times and an average ROC score was calculated to compute how efficient the classifiers were (ROC score: 0.988). After training with the full training set, melting curves were predicted by our tool. Although the ROC score obtained during the evaluation was very high, we wanted to keep a degree of manual inspection as an additional control step. In addition to the “good” or “bad” labelling, our tool calculated the probability of prediction of each curve computed by the SVM. If this probability was below the mean minus one standard deviation of all the probabilities of prediction, the label of the curve became “questionable”, which means it had to be inspected manually. All other curves were labelled according to the SVM prediction in “good” (specific amplification) or bad (unspecific amplification, even if Cq <30 were scored).

### Biomarker signature

To determine whether signatures of miRNAs could classify patients with respect of the different stages, 9 stage comparisons were investigated: (a) healthy versus melanoma, (b) healthy versus stage 0, (c) healthy versus stage I, (d) healthy versus stage II, (e) healthy versus stage III, (f) healthy versus stage IV, (g) healthy versus stages 0+I+II, (h) healthy versus stages III+IV, (i) stages 0+I+II versus stages III+IV. For each of them, a dataset containing only patients at those stages was built. When a group contained more than one stage, the different stages were designated by a common label, e.g. 0+I+II become “early stage”.

For each comparison, to find the signature that had the best stage discrimination, the following procedure was followed: miRNAs were ranked with respect to their importance in stage classification by a SVM classifier, according to the SVM-RFE method [56]. Then, signatures from 2 to 10 miRNAs were built by adding one miRNA, by order of discriminative power, to the signature (e.g. a signature of 2 miRNAs contained the most discriminative miRNA and the second most discriminative miRNA, a signature of 3 miRNAs contains the previous signature plus the third most discriminative miRNA according to the SVM-RFE).

The ability of each signature to classify patients by stage was evaluated through a leave-one-out procedure, i.e. each patient of the set was predicted once by using all other patients as training set for a SVM classifier. The accuracy of classification was determined by counting the number of correct assignments out of the number of patients in the set. The signature with the highest accuracy was considered as best miRNA signature. Accuracy values are indicated for information in Fig. 4B. for this signature a ROC curve was determined by taking half of the patients as training set for a SVM classifier (the other half is used as test set). Note that ranking by the SVM-RFE algorithm is specific to each training set. Combined datasets (e.g. S III+IV) may yield different information when compared to individual datasets. All SVM training and classification have been made using the R package (e1071) and ROC curves have been generated with the pROC package [57].

### Visualization

The heatmaps were created using heatmap.2 function from R Bioconductor package gplots. For generating heatmaps expression values were log2 transformed. MiRNAs that were absent in all samples are not depicted. Principal component analysis (PCA) was performed using R Bioconductor. t-SNE dimensionality reduction [58] was performed using the tsne package from R Bioconductor specifying the perplexity parameter to 10 and using the whiten option.

## SUPPLEMENTARY MATERIAL, FIGURES, TABLES


