# Supplementary material for: Comparison of a healthy miRNome with melanoma patient miRNomes: are microRNAs suitable serum biomarkers for cancer?
Source: Oncotarget. 2015 Mar 26;6(14):12110–27. doi: 10.18632/oncotarget.3661 (PMC4494926; doi:10.18632/oncotarget.3661)
Supplement: Supplementary file 1 [file oncotarget-06-12110-s001.pdf]

Comparison of a healthy miRNome with melanoma patient miRNomes: are microRNAs suitable serum biomarkers for cancer?

Supplementary Material

Supplementary Figure S1: Setup and quality control experiments

A) Plasma versus serum

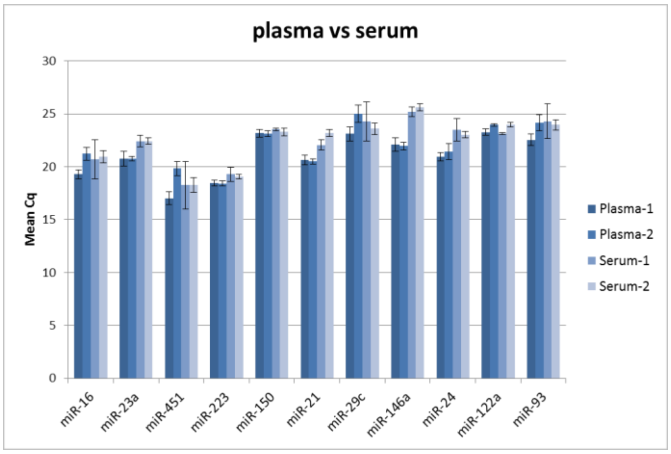

B) Spike-in ratios

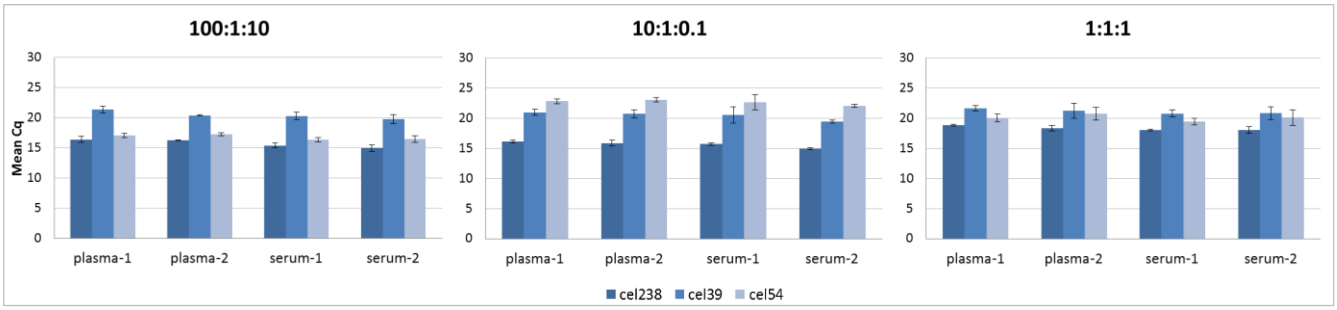

C) Different RNA carriers

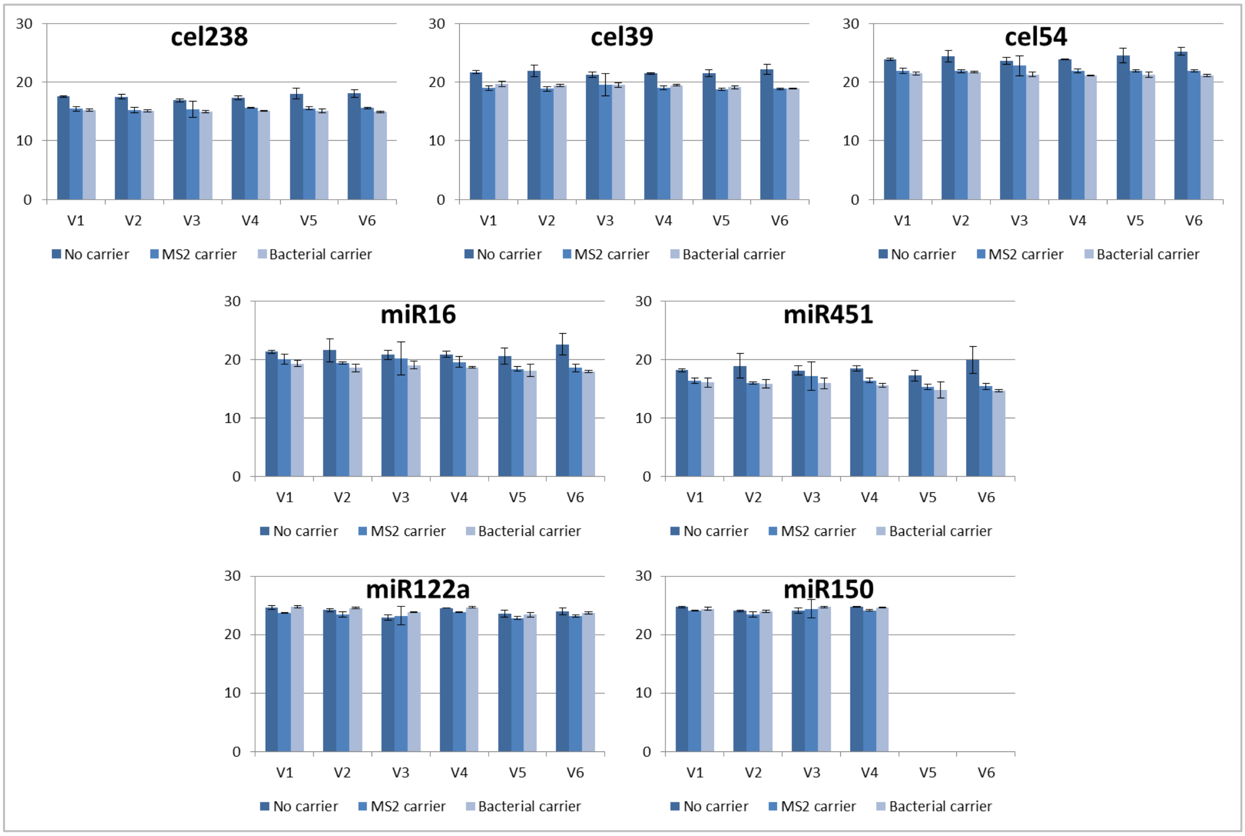

Supplementary Figure S1: Setup and quality control experiments

D) PreAmp vs no PreAmp

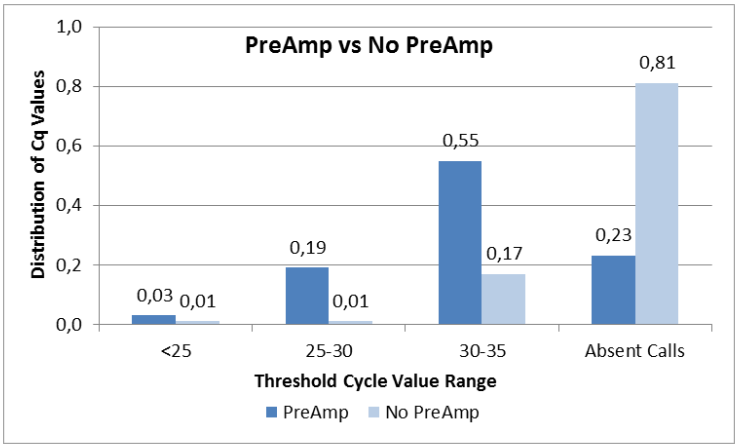

E) PreAmp amplification factor

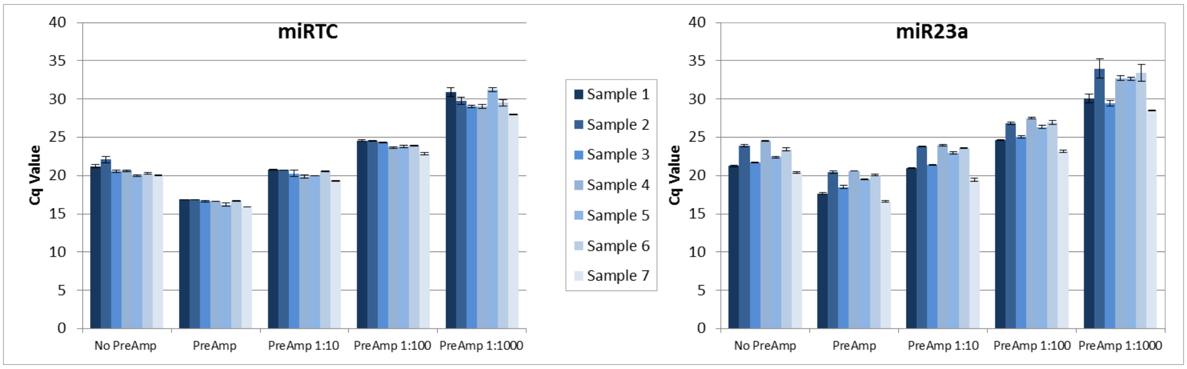

F) Comparison of manual vs whole miRNome qPCR

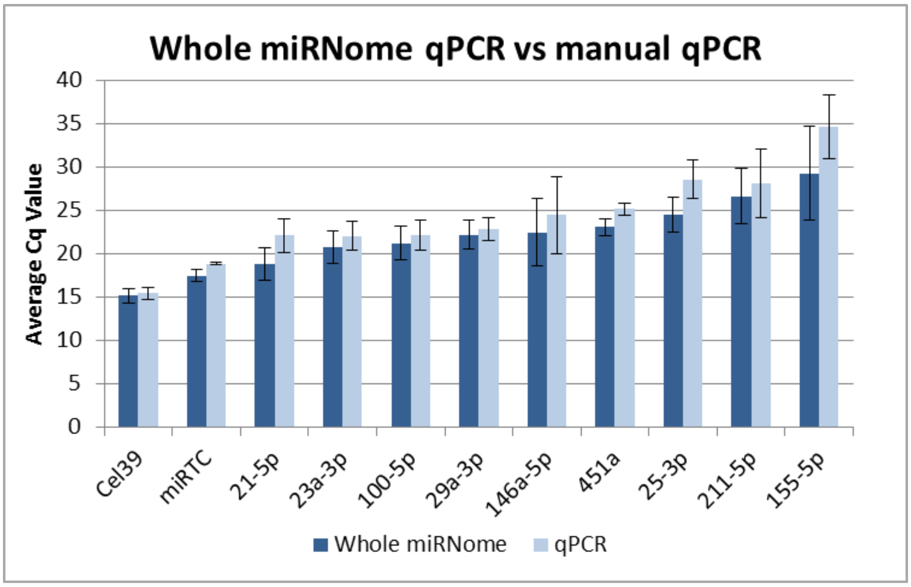

Supplementary Figure S1: Setup and quality control experiments

Setup and quality control experiments.
A) Expression of 11 miRNAs described to be commonly expressed in serum and plasma was analysed in serum and plasma samples. Some, but not all primers showed slightly lower Cq values in the plasma samples. But in general, amplification of serum and plasma miRNAs was quite balanced. Data shown are for 2 samples, but the actual experiment was performed with 4 samples (each serum and plasma).

B) Expression levels of 3 different ratios of the exogenous spike-in controls cel-238, cel-39 and cel-54 were analysed in 2 distinct serum and plasma samples. When equal amounts of the different spikes were added (1:1:1), we observed imbalanced amplification and 100-fold amounts (100:1:10) were too high and amplification reached a plateau. We chose the ratio 10:1:0.1 (cel-238:cel-39:cel-54) for all subsequent experiments. No differences were observed between serum and plasma samples. 0.1: 4.8 x 10^7 copies/µl, 1: 4.8 x 10^8 copies/µl, 10: 4.8 x 10^9 copies/µl, 100: 4.8 x10^{10} copies/µl. Data shown are for 2 samples, but the actual experiment was performed with 4 samples (each serum and plasma).

C) Since serum and plasma expression levels of miRNAs are quite low, different RNA carriers (RNA from bacteriophage MS2 (Roche) and bacterial ribosomal RNA (Roche) were tested during RNA extraction to enhance RNA pulldown. Expression levels of the spike-in controls, of 2 miRNas expressed at higher levels (miR-16-5p and miR-451a) and of 2 miRNAs expressed at lower levels (miR-122-5p and miR-150-5p) were evaluated in the presence or absence of the respective carriers. Regarding spike-in controls and miRNAs expressed at higher levels, both carriers worked more or less equally well to enhance pulldown during RNA extraction (+/- 2 Cqs difference); regarding miRNAs expressed at lower levels, both carriers had no effect. The same effect was observed with 9 more miRNAs (data not shown). Addition of an RNA carrier during extraction seems to introduce a bias toward highly expressed miRNAs. For this reason we did not use RNA carriers for any subsequent experiments.

D) Comparison of positive calls on whole miRNome qPCR arrays from non pre-amplified and pre-amplified cDNA from a single donor. Non pre-amplified cDNA showed 2% of calls within the limit of qPCR detection (0-30 Cq values) whereas pre-amplified cDNA displayed 22% positive calls.

E) Determination of the amplification factor introduced by the pre-amplification step. The Cq values for miRTC and miR-23a-3p of non-pre-amplified cDNA (No PreAmp) were compared to those of 1:25 diluted pre-amplified cDNA (PreAmp) and 10-fold serial dilutions thereof. The Cq values of non-pre-amplified cDNA were equal to those of 1:10 diluted PreAmp sample (1:25 x 1:10 = 1:250), indicating a 250-fold increase in miRNA amount introduced by the pre-amplification step.

F) Reproducibility of whole miRNome qPCR arrays by manual qPCR. Comparison of amplification for 9 miRNAs and 2 controls between whole miRNome qPCR arrays (384-well) and manual qPCR (96-well) using the same amount of input and reaction volume as well as same reagents and cycling conditions showed no significant differences in the Cq output between the two profiling techniques.

Supplementary Figure S2: Heatmaps of all healthy and patient serum samples analysed by custom qPCR arrays

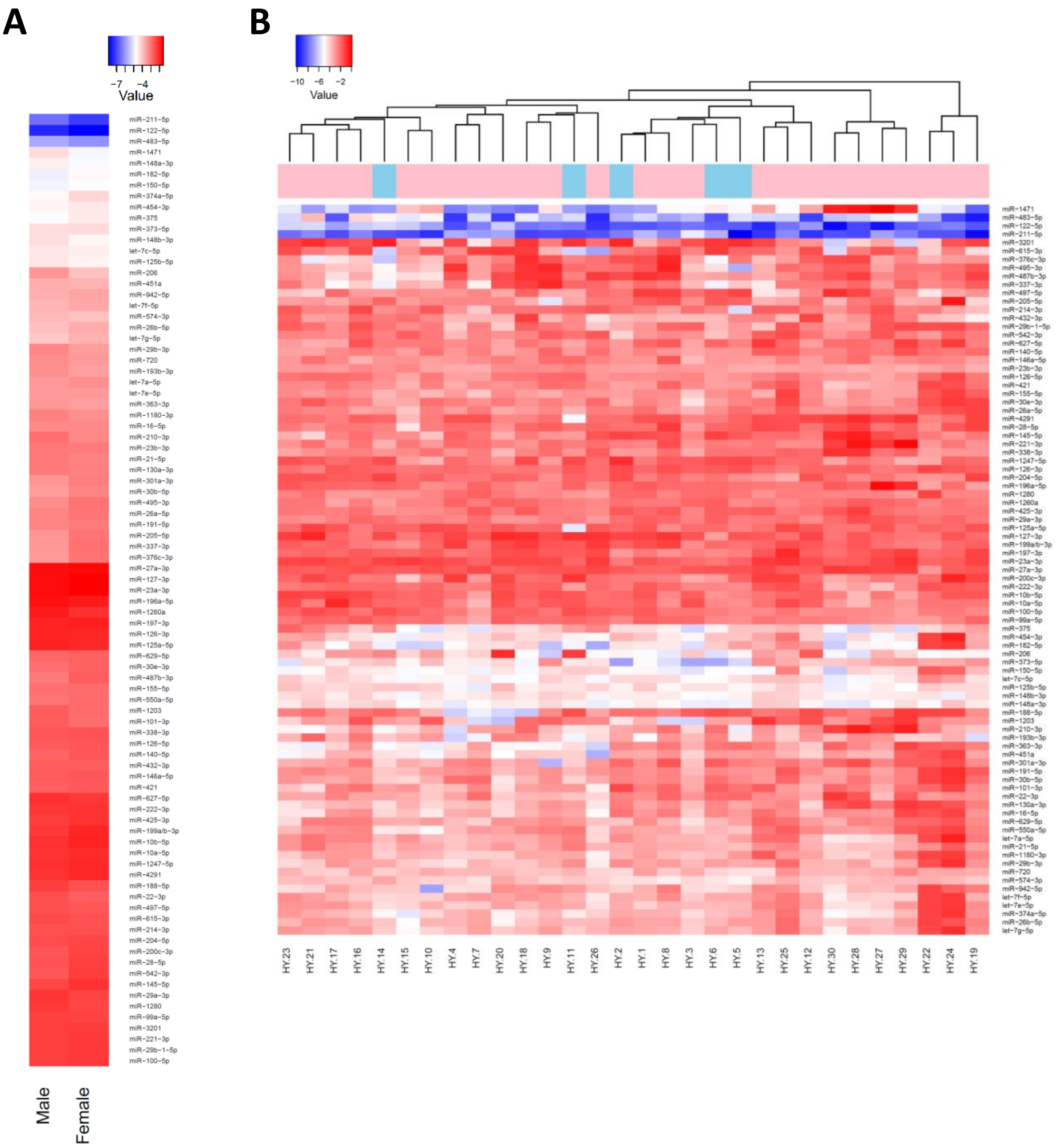

Heatmaps of all healthy and all patient samples analysed by custom qPCR arrays.
Heatmap with the same input file as in Figure 3B. A) Gender differences. Male and female healthy and patient samples were averaged and analysed. No major gender differences were detected. B) Only data of healthy volunteers are depicted; C) only data of patients are depicted. Gender (blue: male, pink: female) and stages of disease are marked (bright to dark: stage 0 to stage IV).

Supplementary Figure S2: Heatmaps of all healthy and patient serum samples analysed by custom qPCR arrays

C

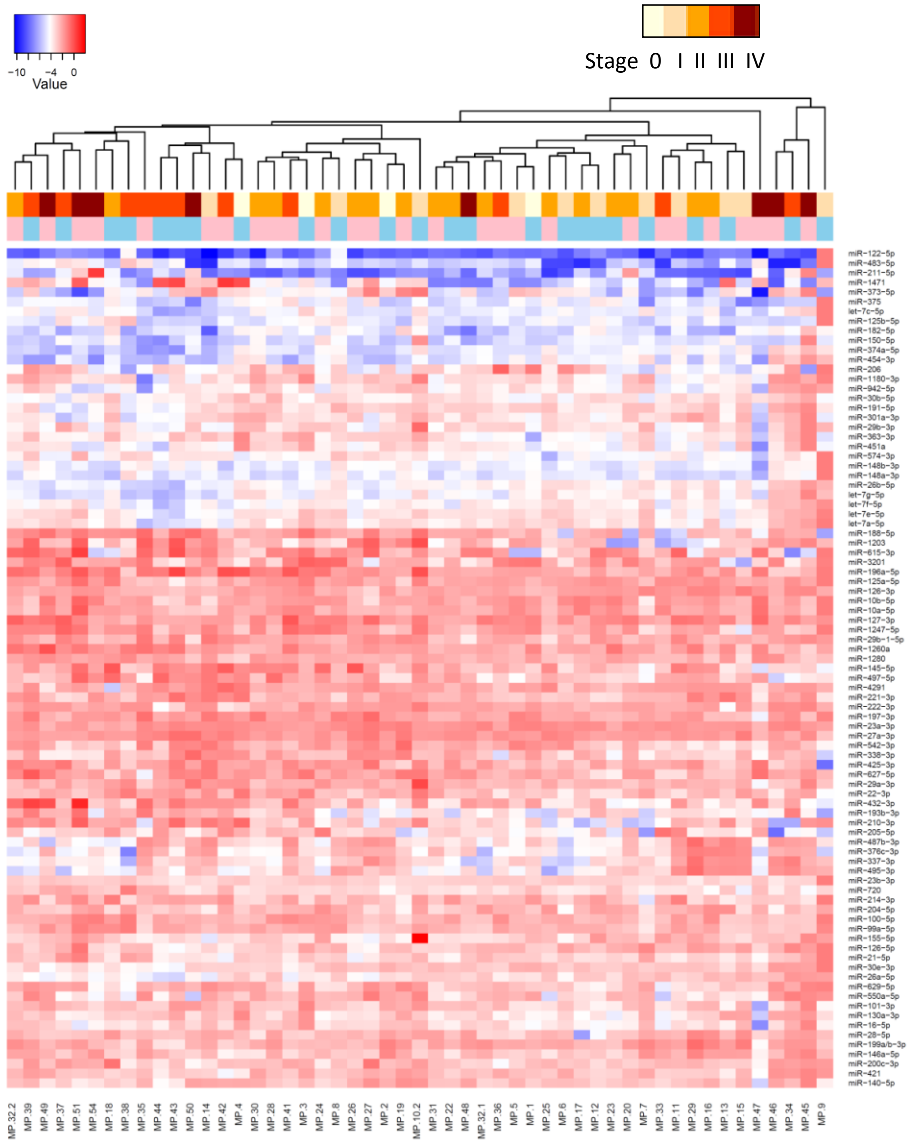

Heatmaps of all healthy and all patient samples analysed by custom qPCR arrays. Heatmap with the same input file as in Figure 3B. A) Gender differences. Male and female healthy and patient samples were averaged and analysed. No major gender differences were detected. B) Only data of healthy volunteers are depicted; C) only data of patients are depicted. Gender (blue: male, pink: female) and stages of disease are marked (bright to dark: stage 0 to stage IV).

Supplementary Figure S3: Heatmaps showing expression of miRNAs of 4 different patients from serum, normal skin and melanoma tissue samples compared to the healthy miRNome.

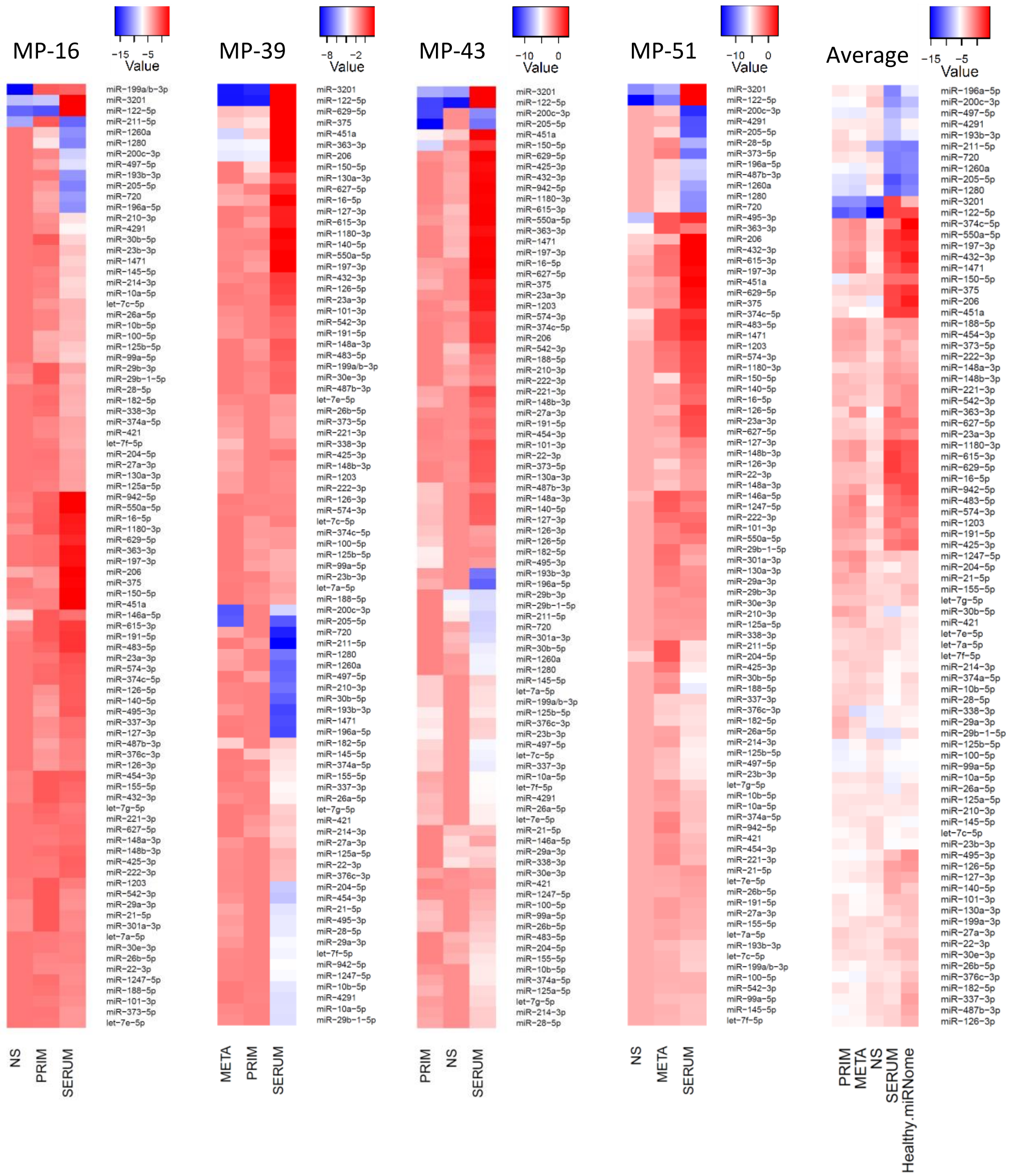

Heatmaps showing expression of miRNAs of 4 different patients from serum, normal skin and melanoma tissue samples compared to the healthy miRNome.
For patient serum samples, data were generated on the custom arrays and for tissues and the healthy miRNome, expression values of the corresponding miRNAs were extracted from the whole miRNome qPCR array data. Data were normalised with the 5 most stable miRNAs calculated for each sample. Far right: heatmap on averaged data for sample types of the 4 patients including the healthy miRNome. NS: normal skin, PRIM: primary melanoma tissue, META: metastatic tissue. Note that color scales are not the same for all heatmaps.
